# Diphtheria: Its Nature and Treatment

**Published:** 1889-07

**Authors:** 


**Diphtheria: Its Nature and Treatment.** By C. E. Billington, M. D.; and **Intubation in Croup** and other acute and chronic forms of stenosis of the larynx. By Joseph O'Dwyer, M. D. 8vo, pp. iv., 326; cloth, illustrated. New York: William Wood & Co. 1889.

Until the results in the treatment of diphtheria show a higher percentage of recoveries than at present, no excuse is necessary from an observer of large experience for again calling attention to the subject. Dr. Billington has had an extensive experience with this disease, and is, moreover, a close and accurate observer, and this work from his pen is, therefore, an important contribution. It has many points of excellence. It is comprehensive, and quotes impartially and at length all who have added anything of moment to the knowledge of the disease. It is judicious, and carefully refrains from accepting conclusions from insufficient data, or those based upon the observations of limited opportunity. It is critical, and analyzes with great force the various opinions in regard to its nature, etiology, and treatment. Naturally, the author has come to hold definite views upon some of the disputed points, but he states his own opinions with fairness and an appreciation of the work of those from whom he differs.

The conclusions in regard to the etiology of diphtheria, which he considers as probable, are as follows:

"1. Diphtheria is caused by a parasite which has the following characteristics: Its growth and multiplication outside of the body are favored by dampness and insanitary conditions, and it is reproduced in the disease; its presence on mucous membranes is sometimes innocuous; its vital activity is greatly increased under the conditions which prevail during an epidemic; its pathogenic action is greatly favored by preëxisting morbid conditions of the body, and especially those involving lesions of the epithelium; it is transmitted from one person to another by the various processes which are most usually included under the terms contagion and infection.

2. This parasite causes diphtheria by being planted on a mucous membrane or wounded surface of the body, or in its more superficial tissues, and there producing a chemical poison or ptomaine.

3. This poison or ptomaine, by its direct action on the tissues and vessels, causes the local diphtheritic process, in the course of which it is reproduced and more and more widely diffused, and, by its absorption from this source into the general circulation, causes the constitutional disease.

4. This morbid process is often accompanied or followed by the invasion of the body by other pathogenic bacteria, to which various complications are due.

5. No bacterium thus far discovered in connection with diphtheria can furnish by its presence or its absence a reliable criterion for diagnosis."

It will be seen from the above that the author believes that diphtheria is primarily a local disease. This position is further sustained by the consideration of the following points:

"1. Diphtheria occurs in the great majority of cases upon the mucous membrane of the fauces, the larynx, the nasal passages and the mouth, or, in other words, the outer avenues of entrance of inspired air and of food and drink, and with the greatest relative frequency in exactly those positions where particles of matter introduced by them would most naturally be deposited, which fact suggests a probability that the disease is directly and locally caused by such contact or implantation.

2. The constitutional disease is, in the order of time, not antecedent to, but consequent upon, the local affection.

3. The gravity of the general disease varies directly, as a general rule, in proportion to the extent and, more particularly, the depth of the local affection.

4. The employment of proper local antiseptic treatment does in many cases promptly mitigate or quite dispel constitutional symptoms previously existing, or, if early employed, prevent either wholly, or in some measure, their occurrence, and its failures to accomplish these objects occur in exactly those cases in which, from the nature of things, it cannot be, or in which it is not efficiently employed."

These points are discussed in detail and in a way to carry conviction to most unprejudiced minds.

In regard to diagnosis, Dr. Billington holds, correctly we believe, that follicular tonsillitis is not a mild diphtheria, but a totally distinct disease. He reviews this subject at some length, and considers the various questions connected with it in the most satisfactory manner. The whole chapter on Diagnosis is a valuable part of the book.

We have referred to a few of the disputed questions concerning this disease, and have quoted the author's opinions in regard to them, that the reader might have some idea of the thoroughness with which Dr. Billington handles the subject.

The chapter on Treatment will naturally attract considerable attention. It is exhaustive, and we cannot do more at this time than refer to its general arrangement. It is divided into sections under the following heads: General Indications, General Principles of Treatment, the Results of Treatment, the Modes of Employing Remedies, Caustics, Astringents, Agents for the Destruction of False Membranes, Solvents of False Membrane, Antiseptics (discussing the various drugs possessing the qualities of an antiseptic that have been used in the disease), Specifics, Cardiac Depressants, the Treatment of Diphtheria by Irrigation, and, finally, the Method of Treatment which has been Employed by the Author.

Tracheotomy is then considered, and afterward the treatment of the various sequelæ. This chapter must be read by all those who desire to be familiar with best treatment of diphtheria, as well as with the various methods that have been suggested.

The second part of the book considers Intubation, and is from the pen of the inventor of this most valuable proceeding, Dr. Joseph O'Dwyer. A great deal has been written upon this subject without exhausting it, and we welcome this contribution as containing the latest opinion and criticisms of this method. Dr. O'Dwyer shows that he is imbued with the truly scientific spirit of inquiry. He adopts all suggestions that have been proved of value in regard to intubation. He discusses the method of procedure, the accidents attending it, the results following it, and all other questions that may arise concerning it, in a most clear and concise manner.

Tracheotomy and Intubation are credited with raising the recoveries from fibrinous croup about 27 per cent., a result which establishes their position beyond all question. Which method to employ in any particular case, must be decided by the consideration of all the circumstances surrounding it, and cannot be determined beforehand.

The book is well printed. It should be owned by all who have to deal with diphtheria in any way, for we believe a careful study of its pages will be of assistance even to those who already have a large experience in the treatment of this disease. Those who have not had this experience will find it almost invaluable.

